# Reducing research waste by promoting informed responses to invitations to participate in clinical trials

**DOI:** 10.1186/s13063-019-3704-x

**Published:** 2019-10-28

**Authors:** Katie Gillies, Iain Chalmers, Paul Glasziou, Diana Elbourne, Jim Elliott, Shaun Treweek

**Affiliations:** 1 Health Services Research Unit, University of Aberdeen, Aberdeen, AB25 2ZB, UK; 2 Centre for Evidence-Based Medicine, University of Oxford, Edinburgh, UK; 3 Bond University, Robina, QLD 4226, Australia; 4 London School of Hygiene and Tropical Medicine, Keppel Street, London, WC1E 7HY, UK; 5 Health Research Authority, Skipton House, London, SE1 6LH, UK

**Keywords:** Recruitment, Retention, Clinical trials, Research waste, Informed choice

## Abstract

Poor recruitment to, and retention in, clinical trials is a source of research waste that could be reduced by more informed choices about participation. Barriers to effective recruitment and retention can be wide-ranging but relevance of the questions being addressed by trials and the outcomes that they are assessing are key for potential participants. Decisions about trial participation should be informed by general and trial-specific information and by considering broader assessments of ‘informedness’ and how they impact on both recruitment and retention. We suggest that more informed decisions about trial participation should encourage personally appropriate decisions, increase recruitment and retention, and reduce research waste and increase its value.

## Background

Poor recruitment to, and retention in, clinical trials can be important sources of waste in clinical research [1–4]. This source of waste may result from, for example: regulatory barriers; inappropriate study design, particularly inclusion criteria; failure to use effective and efficient recruitment and retention strategies; negative attitude of patients and clinicians to trials; and the (ir)relevance of the study question to patients and clinicians.

The reasons for poor recruitment to, and retention in, clinical trials need to be diagnosed so that appropriate strategies for prevention and treatment can be implemented. Evidence for effective strategies is currently limited: the Cochrane recruitment review, for example, identified 72 wide-ranging recruitment interventions, but only three of them (open vs. blinded trials; telephoning non-responders; a particular, structured process for developing participant information leaflets) are supported by high-certainty evidence to improve recruitment [5]. The sister Cochrane review on strategies to improve retention identified more than 50 interventions (the majority focussing on improving questionnaire return), of which only monetary incentives were shown to be effective [6]. Investigating how interventions to improve recruitment influence retention was evaluated by only one trial included in both of these reviews [7].

Informed agreement to participate and stay in a clinical trial is unlikely if the research question it addresses, the interventions it is comparing and the measures of treatment outcomes planned are of little or no interest to potential participants and their clinicians. Four systematic reviews [8–11] have found perceived personal benefit to be a key motivator for taking part in a trial: if it is not clear that a trial has at least the prospect of benefit, potential participants are unlikely to become actual participants. By contrast, if the questions addressed and the trial’s design have been chosen by a multidisciplinary team, including patients and healthcare professionals [12], it is reasonable to expect that the study will receive support from potential participants and healthcare professionals. Priority setting exercises involving patients, healthcare professionals, and other relevant stakeholders are now becoming a widely accepted method in health services’ research to identify and prioritise research questions in key clinical areas (http://www.jla.nihr.ac.uk/about-the-james-lind-alliance/about-psps.htm). These ‘Priority Setting Partnerships’ ensure relevance, acceptability, and importance of questions to all stakeholders.

For example, people with asthma and the clinicians caring for them agreed some years ago that it was important to address uncertainties about the value of breathing-retraining programmes [13]. An appropriately designed randomised trial was commissioned to address this uncertainty. The trial recruited the intended number of participants within the planned timescale and showed that breathing-retraining programmes improve quality of life and that they can be delivered cost-effectively using self-guided audiovisual media [14]. The Football Fans in Training trial also recruited to target and retained well (92%), in part because of the involvement of future users of the results – football clubs – in design decisions [15].

## Informed consent to participate in clinical trials

A book about clinical research written for the public (‘*Testing Treatments*’) advised readers only to accept invitations to participate in clinical trials if the researchers could show that they were addressing important uncertainties [16]. There are two components to important uncertainties: (1) that systematic reviews of all current relevant research have shown substantive residual uncertainties about the effects (benefits or harms) of the treatment(s); and (2) that resolving those uncertainties would be important to a body of patients and clinicians. It is clearly not in the public interest to increase recruitment to clinical trials addressing questions that are not important to patients [17]. This implies that potential participants in clinical trials need to be discerning when deciding which trials to support. To achieve this, they need to have both general (related to the trial’s endeavour more broadly) and specific knowledge (of relevance to the particular trial for which they are considering participation).

The general knowledge needed by potential participants is an awareness of the damage that has been done by the continued use of inadequately tested treatments in the past, and that this remains a problem with today’s treatments [16].

The specific pieces of information needed by potential participants in a particular trial are the current basis of the treatment(s) and the residual uncertainties as demonstrated by systematic reviews of all previous relevant research about their effects (benefits or harms). Whilst there is currently a lack of empirical evidence about what information potential participants want when facing a decision about participation and even less about what they want during the research [18], the exact content of this information should be decided and co-produced with patient and clinician partners during trial design. Table 1 lists both general and specific questions which may be relevant to ensuring informed decisions about participation in clinical trials.

**Table 1:** Questions identifying general and specific information needed to make informed choices about participating in a trial

General	
• Do they know that new treatments are about as likely to be worse as they are to be better than alternative existing treatments [17]?	
• Do they understand why random allocation to treatment comparison groups is used in ‘fair tests’, and why blinding outcome assessment is desirable if possible [15]?	
• Are they aware of the importance of providing required outcome data to contribute to analysis?	
• Are they aware that insufficiently large studies and the failure to report some studies have resulted in lethally mistaken beliefs about the effects of treatments [15]?	
Specific	
• Are potential participants told whether similar patients to them have contributed to the design of the trial?	
• Are the questions being addressed by the trial relevant to their personal interests?	
• Are they supported to make decisions appropriate for them as individuals?	
• Are they told about the requirements of the trial as a whole (i.e. the work associated with taking part and completing the trial) and not just the recruitment process?	
• Are they satisfied that those collecting, analysing and interpreting the evidence yielded by the trial are sufficiently free of competing interests?	
• Have they been assured that a full report of the trial will be published?	

## Conclusions

Can potential trial participants be properly informed without having general and trial-specific knowledge?

Evidence from a systematic review highlights a lack of understanding on general items (e.g. randomisation, voluntariness) and trial-specific items (e.g. overall aim, treatment risks and benefits) amongst research participants [19]. Yet, participant-reported measures of informed consent often do not attend to assessments that go beyond understanding – leaving unanswered the question of whether consent was an informed decision [20]. What seems to be missing is a body of evidence derived from observing interactions between trial recruiters and potential participants, and information about whether both parties have a good grasp of the key concepts needed to support informed choices [21]. The Quintet Recruitment Intervention recommends recording conversations between recruiters and potential participants to learn more about the recruitment conversation and how that conversation could be improved [22]. However, it should be noted that requests to record these conversations have sometimes been rejected by a substantial minority of potential trial participants [23].

Research on participant information leaflets, a key part of the recruitment process, has found that most leaflets do not provide the information needed to support informed decision-making [24], and the same may well be true of recruitment discussions. Preliminary work to develop and evaluate decision aids (tools that aim to support informed choices about options) for trial participation has shown promise by supporting decisions that align with individual’s values and expectations [25–27]. Whether and how this translates to overall improvements in recruitment and retention requires further investigation. Work is also underway to explore and agree which outcomes to use to evaluate attempts to improve the consent process [20]. Consent seems increasingly likely in future to be sought using digital media, a development that will offer both challenges and opportunities [28].

Researchers and clinicians could engage more effectively with patients and the public to promote trials. Evidence from a recent survey revealed that only 37% of the public said that they trusted evidence from medical research [29]. This finding may be one of the reasons for poor recruitment in clinical trials and the resulting waste of resources. A potential opportunity to address this lack of trust (and other improvements in recruitment and retention) could lie in bringing together activated, empowered, patient groups through campaigns such as #wearenotwaiting [30]. This group of activated patients could act as peer educators allaying myths and misunderstandings about trials generally within patient communities. International efforts to improve Patient and Public Involvement (PPI) are also gathering speed with the development of initiatives such as the #globalPPInetwork. A key priority emerging from the latter is the need to develop training particularly for patients and the public working on clinical trials [31].

There are already relevant learning resources available, for example, those made available through www.testingtreatments.org, and research has shown that it is possible to teach primary school children and adults to apply some of the key concepts needed to inform treatment choices [32, 33]. Wider acquisition of these skills should help people to make informed treatment choices, but it may also help them to make more informed decisions about whether to participate in clinical trials when there are uncertainties about the relative merits of the different treatment options open to them.

The need for informed decision-making in response to invitations to participate in clinical trials remains when a decision to take part is made on behalf of an individual (e.g. because of cognitive impairment) or on behalf of a population (e.g. a regional evaluation of a screening intervention). In these circumstances it falls to staff at the institutions invited to participate in research to ensure that they are aware of the evidence that proposed studies will address confirmed, important uncertainties about the effects of treatments [34]. They too need both general and trial-specific knowledge to make their decisions.

Potential participants in clinical trials need to be appropriately informed and discerning when deciding which trials to support. Informed decisions seem likely to promote successful recruitment and retention, and thus to reduce waste in clinical research. Public capacity to make sufficiently informed decisions will depend on greater efforts to promote the general knowledge needed to assess evidence of important uncertainties and how they should be addressed. This may be achieved, for example, through raising public awareness of trials and fostering critical appraisal of evidence. Also needed are better means to provide the specific knowledge required for decision-making about individual trials, which will likely require development of decision-support interventions. Whilst researchers will be key in realising these benefits, failure to involve patients and healthcare professionals may result in avoidable inefficiency and waste in clinical trials.

## Data Availability

Not applicable.
